# Effectiveness of Hospital-Based Systemic Rehabilitation in Improving Ankle Function after Surgery in Chronic Ankle Instability Patients

**DOI:** 10.1155/2021/6695096

**Published:** 2021-01-28

**Authors:** Da Hye Kong, Gun Sang Lee, So Hee Park, Min Cheol Joo, Sung Hyun Lee, Min Su Kim

**Affiliations:** ^1^Department of Rehabilitation Medicine, Wonkwang University College of Medicine and Institute of Wonkwang Medical Science, Iksan, Republic of Korea; ^2^Department of Orthopedic Medicine, Wonkwang University College of Medicine and Institute of Wonkwang Medical Science, Iksan, Republic of Korea

## Abstract

We investigated the therapeutic effect of a postoperative hospital-based systemic rehabilitation protocol on ankle function in chronic ankle instability (CAI) patients. Thirty-five patients who underwent a modified Broström procedure for CAI were recruited in this prospective randomized controlled trial. Fifty-minute sessions of hospital-based rehabilitation were performed three times weekly for 12 weeks in the intervention group. Education-based rehabilitation was conducted at home in the control group. The outcomes were evaluated at baseline (T0), 12 weeks (T1), and 16 weeks (T2). The primary outcome was the foot and ankle outcome score (FAOS). Ankle motor strength and spatiotemporal gait metrics were assessed as secondary outcomes. There were significant time and group interaction effects on the pain, symptoms, activities of daily living, sports activities, and quality of life (QOL) domains of the FAOS (*P* < 0.05, all). The patients in the intervention group showed larger improvements in all domains of the FAOS than did the control group at both T1 and T2 (*P* < 0.05, all). The time and group interaction effects on invertor and evertor strength were also significant (*P* = 0.047 and *P* = 0.044). Invertor and evertor strength improved significantly more in the intervention group than in the control group at T1 and T2 (*P* < 0.05, all). The preferred walking velocity, cadence, step length on the affected side, and double stance phase duration tended to improve over time. Postoperative hospital-based rehabilitation helped improve CAI pain, symptoms, independence in activities of daily living, sports activity levels, and QOL more effectively than did conventional rehabilitation at home.

## 1. Introduction

Damage to the lateral ankle ligaments by forced inversion of the ankle joint is one of the most common sports injuries [1]. Up to 20% of patients continue to suffer from lateral ankle instability, characterized by recurrent ankle sprains or a feeling of apprehension in the ankle [2]. Chronic ankle instability (CAI) is defined as a frequently recurring feeling over a period of six months of the ankle giving way due to an unstable ankle movement pattern similar to an ankle sprain [3]. The initial treatment of chronic ankle instability is usually rehabilitation therapy, consisting of several training programs; however, surgery should be considered if symptoms persist [4]. The Broström procedure, which has been modified several times, has been considered the gold standard of surgical treatment for several years [5, 6].

Even after the modified Broström procedure (MBP) is performed successfully, several patients persistently develop complications such as pain, ankle contractures, and recurrent instability [7]. It has been reported that approximately 9% of patients demonstrate severe ankle stiffness, and ankle instability persists in 5-12% of patients after the MBP [7, 8]. Systemic rehabilitation therapy can help CAI patients return early to daily activities by minimizing the risk of postoperative complications and reinjury [9]. Postoperative rehabilitation typically consists of joint mobilizing exercises, strengthening exercises, proprioceptive training, and balance training, which can optimize lower limb postural control and restore stability [10–13]. Tape and braces are also commonly used to provide external mechanical support as well as enhance proprioception [2]. Although rehabilitation programs vary slightly depending on the surgeon, joint exercises and partial weight-bearing training are usually started soon after the MBP, and neuromuscular exercises and gait training are gradually started as the patient's capabilities progress [14–16].

However, many CAI patients commonly conduct postoperative rehabilitation exercises at home by themselves without any supervision after a simple guided session, and some patients receive training from nonprofessionals at a gym. Inappropriate rehabilitation in the postoperative stage for patients such as CAI patients may result in unsatisfactory outcomes, even when the MBP is performed successfully. We established a hospital-based systemic rehabilitation protocol for CAI patients to perform after ankle surgery to solve these problems. The rehabilitation protocol was conducted in close collaboration with orthopedic surgeons. Physiatrists and physical therapists directly performed rehabilitation therapy and monitored the patients in the hospital. The purpose of this study was to investigate whether hospital-based intensive rehabilitation programs are more effective in restoring ankle function than are education-based self-exercises at home in post-MBP patients.

## 2. Materials and Methods

### 2.1. Subjects

We recruited CAI patients with anterior talofibular ligament (ATFL) grade 2 or grade 3 who had undergone the MBP due to unresponsiveness to conservative treatment over three months. An orthopedic professor (MD) specializing in ankles and feet was responsible for all patient surgeries. Severity level of the ATFL was classified by arthroscopic grade based on a previous study: grade 0, normal and continuous ligament without tearing, normal thickness, and taut between the lateral malleolus and the talar neck; grade 1, distended ligament without tearing, normal in thickness but with decreased tension by hook palpation; grade 2, fibular or talar avulsion (with fibrous tissue) of the ATFL, normal thickness, but decreased tension by hook palpation; grade 3, thin ATFL ligament with no mechanical resistance by hook palpation, with or without scar tissue; and grade 4, scar tissue with absent ATFL [17]. Arthroscopic grade 2 or 3 CAI patients underwent MBP, and the orthopedic surgeon referred them to the Department of Rehabilitation Medicine in a tertiary hospital to perform rehabilitation therapy on an outpatient basis.

All patients received the routine immediate postoperative program. A compression bandage and a posterior plaster splint were applied to the ankle in a neutral position and were maintained for 2–3 days after the surgery. Thereafter, a removable walking boot was used for 4 weeks. Partial weight-bearing was allowed with an assistive device in the first 2 weeks. Full weight-bearing and active range of motion exercises were permitted from 4 weeks after the surgery.

We recruited CAI patients who had undergone the MBP due to unresponsiveness to conservative treatment for three months. The orthopedic surgeon referred the participants to the Department of Rehabilitation Medicine in a tertiary hospital to perform rehabilitation therapy on an outpatient basis. The participants began therapy an average of approximately four weeks after MBP. Participants who had (1) a severe injury on the opposite lower extremity as well or (2) a history of an ankle fracture or bony malalignment, and (3) major cardiovascular, psychiatric, or musculoskeletal diseases were excluded from this study.

### 2.2. Randomization and Masking

This study was designed as a prospective randomized controlled trial with two arms. A Study Collaborating Studies Coordination Center of Wonkwang University used randomization to allocate patients to groups. This allocation procedure was concealed from clinicians. The database is independent and thus is not accessible to study personnel except to receive a specific patient treatment assignment. Participants were randomly assigned 1 : 1 to receive hospital-based rehabilitation with the supervision of trained physical therapists or to perform self-rehabilitation at home. The Research Randomizer (https://www.randomizer.org/), which generates a predefined randomization list, was used to assign patients to each group. All investigators were masked to group assignment for the trial duration, including the evaluation of all data and outcomes.

### 2.3. Surgical Procedure

For arthroscopic repair, a medial midline portal (immediately lateral to the tibialis anterior tendon) was used as the viewing portal. An accessory anterolateral (acAL) portal was used for the working portal. Arthroscopic ATFL repair was performed using a knotless anchor (PushLock 2.9 mm × 15 mm; Arthrex, Naples, FL) by shuttle relay method with a suture passer (Micro SutureLasso, curved 70°; Arthrex, Naples, FL). After ATFL anatomical repair, another suture anchor (SutureTak, 3.0 mm × 14.5 mm; Arthrex, Naples, FL) with two FiberWire pairs was introduced to the proximal aspect through the previous anchor from the acAL portal. After one limb of each paired FiberWire was passed through the inferior extensor retinaculum (IER), the two FiberWire pairs were tied using the Samsung Medical Center sliding knot technique augmentation of the IER at the distal fibula.

### 2.4. Intervention

This study was designed as a prospective randomized controlled trial with two arms. The participants were randomly assigned into two groups: an intervention group that received hospital-based rehabilitation with the supervision of trained physical therapists and a control group that performed rehabilitation independently at home. The interventions continued for twelve weeks.

The patients in the intervention group visited the physical exercise room in the participating hospital three times a week for 50 minutes and received rehabilitation therapy with the supervision of physical therapists. The rehabilitation program was composed of a joint range of motion exercises, proprioceptive training, strengthening exercises, and balance training to improve ankle function. The exercises were adjusted over time according to the following three stages: the early (postoperative 4-6 weeks), middle (postoperative 8-10 weeks), and late (postoperative 12-14 weeks) stages.

In the early stage, rehabilitation consisted of preparatory exercises, weight-bearing exercises, and strength training. Passive joint exercises and ankle pump exercises were performed as preparatory exercises, and weight shifts to the affected side was attempted repeatedly. Every three sets of active dorsal and ventral flexion exercises of the ankle joint were performed using elastic resistance bands. Additionally, three sets of a leg-raise exercise and bridge exercise were conducted to strengthen muscles in the lower extremity other than those spanning the ankle. In the middle stage, rehabilitation therapy was performed to restore full range of motion in the ankle joint, muscle strength to as high as 70% of the average level, and proprioceptive sensation. Aerobic exercise using a stationary bicycle was performed for 10 minutes, and the ankle range of motion was gradually increased. Ankle invertor/evertor exercises using an elastic resistance band and one-leg standing/lunge exercises were performed to promote strength recovery and active joint mobilization. If the patients could maintain balance for 30 seconds, a balance board standing trial with one leg was attempted. In the late stage, all these training components were integrated, and more challenging versions were performed by the patients. Active ankle joint mobilization was promoted until full range of motion was reached, and treadmill exercise was performed for 10 minutes. Three sets of lunges with 3 kg dumbbells and squats were conducted, and one-leg hopping was added to promote balance.

The patients in the control group attended one educational session to learn how to perform the same rehabilitation protocol at home. A picture-guided booklet was provided to ensure that they could conduct the protocol appropriately at home. All the participants visited the outpatient clinic for orthopedic surgery and rehabilitation medicine every four weeks to ensure that the rehabilitation therapy was being correctly administered in both groups.

### 2.5. Outcomes

All outcome measures were assessed three times: preintervention (T0), postintervention (T1), and four weeks after the end of the intervention (T2). The foot and ankle outcome score (FAOS) was used as the primary outcome to assess ankle function and to compare the therapeutic effects between the two groups. It consists of five subdomains: pain, symptoms, sports and recreational activities (SRA), activities of daily living (ADL), and quality of life (QOL) [18]. The score ranges from 0 (very poor) to 100 points (no symptoms), and we obtained the FAOS by face-to-face assessments [19].

Several measures were included as the secondary outcome measures. Ankle motor strength, including invertor, evertor, dorsiflexor, and plantar flexor strength, were measured using an isokinetic Cybex dynamometer (CSMi, MA). The peak torque of the involved ankle was measured when the participants performed 15 isokinetic cycles at an angular velocity of 180°/second. Spatiotemporal gait analysis was performed using a treadmill system (Zebris, Germany) to investigate the change in walking pattern after the intervention. The preferred walking velocity, cadence, step length, stride length, ratio of stance/swing phase, and double stance phase duration as a percentage of a gait cycle were calculated.

We recorded each patient's age, sex, height, weight, body mass index (BMI), operated side, and period after MBP as the baseline characteristics before starting the intervention.

### 2.6. Statistics

The differences in the baseline clinical characteristics between the groups were analyzed using an independent *t*-test for ordinal variables and the chi-square test for nominal variables. Repeated measures analysis of variance was used to evaluate time and group interaction effects to identify significant differences between the groups in the degree of change over time. A post hoc analysis was performed with Bonferroni's correction. We calculated the change value (*Δ*) of the outcome measures from baseline to each time point postintervention, and an independent *t*-test was applied to compare the degree of change between the groups. All of the data were statistically analyzed using SPSS 22.0 (SPSS, IBM). The level of statistical significance was set to be 0.05.

### 2.7. Ethics Statement

The present study protocol was reviewed and approved by the Institutional Review Board of Wonkwang University Hospital (Approval No. 2019-01-019). All patients provided written informed consent.

## 3. Results

**Figure 1:**
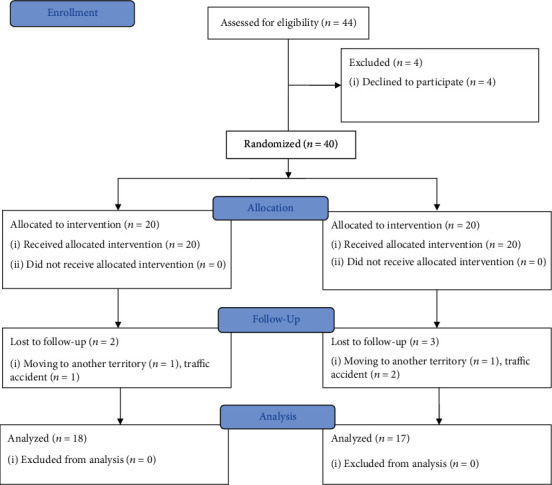
Participants' selection flow.

A total of 44 patients were eligible for this study (Figure 1). Four patients refused to participate in the study, and five patients were lost to follow-up due to traffic accidents and personal reasons. Eventually, the data from 35 patients were analyzed. The participants began rehabilitation at an average of 17.9 ± 4.5 days after the MBP, and the mean age was 39.9 ± 7.1 years old (Table 1). There were no significant differences in baseline characteristics between the intervention and control groups. Additionally, adverse effects such as reinjury, falls, and cardiovascular accidents were not reported in any of the participants.

### 3.1. Primary Outcome Measures

The baseline scores for each FAOS domain did not significantly differ between the two groups (Table 2). There were significant time and group interaction effects on the pain, symptoms, ADL, SRA, and QOL domains of the FAOS (*F*_1,33_ = 5.243, *P* = 0.029; *F*_1,33_ = 4.394, *P* = 0.044; *F*_1,33_ = 4.250, *P* = 0.047; *F*_1,33_ = 15.922, *P* = 0.008; and *F*_1,33_ = 4.016, *P* = 0.048, respectively). In the intervention group, FAOS for pain, symptoms, ADL, SRA, and QOL significantly increased after 4 weeks of the intervention (Δ_T1−T0_ = 14.9 ± 5.2, *P* < 0.001; Δ_T1−T0_ = 15.2 ± 6.3, *P* = 0.002; Δ_T1−T0_ = 14.1 ± 5.8, *P* = 0.005; Δ_T1−T0_ = 31.6 ± 14.2, *P* < 0.001; and Δ_T1−T0_ = 20.7 ± 10.0, *P* < 0.001, respectively). The patients in the control group also showed significant improvements in the pain, symptoms, ADL, SRA, and QOL domains (Δ_T1−T0_ = 5.8 ± 2.5, *P* = 0.020; Δ_T1−T0_ = 4.9 ± 2.4, *P* = 0.012; Δ_T1−T0_ = 6.8 ± 3.9, *P* = 0.001; Δ_T1−T0_ = 8.7 ± 4.3, *P* = 0.028; and Δ_T1−T0_ = 11.7 ± 5.1, *P* = 0.010, respectively). These improvements lasted for 4 weeks in both the intervention and control groups.

Post hoc analysis revealed that the patients in the intervention group demonstrated larger improvements at T1 in the FAOS subdomain scores for pain, symptoms, ADL, SRA, and QOL than did the patients in the control group (*P* = 0.010, *P* = 0.024, *P* = 0.020, *P* = 0.003, and *P* = 0.036, respectively). In addition, larger ankle function improvements in the pain, symptoms, ADL, SRA, and QOL domains were observed in the intervention group than in the control group at T2 (*P* < 0.001, *P* = 0.022, *P* = 0.020, *P* < 0.001, and *P* = 0.032, respectively).

### 3.2. Secondary Outcome Measures

There were no significant differences in the baseline values of ankle muscle strength or the spatiotemporal gait metrics between the two groups (Table 3). The time and group interaction effects on ankle invertor and evertor strength were significant (*F*_1,33_ = 4.521, *P* = 0.047 and *F*_1,33_ = 4.369, *P* = 0.044). Ankle invertor strength increased from 20.5 ± 9.4 to 23.1 ± 9.5 Nm at T1 and to 25.2 ± 10.1 Nm at T2 in the intervention group. In the control group, it increased from 21.1 ± 9.6 to 22.1 ± 9.6 Nm at T1 and to 22.2 ± 10.4 Nm at T2. A significant difference in ankle invertor strength was found between groups at T1 and T2 (*P* = 0.010 and *P* = 0.005). Additionally, ankle evertor strength improved from 16.1 ± 5.4 to 17.6 ± 6.1 Nm at T1 and to 18.0 ± 7.5 Nm at T2 in the intervention group. The control group showed a slight improvement in ankle evertor strength from 15.8 ± 6.0 to 15.9 ± 6.1 Nm at T1 and to 16.0 ± 6.3 Nm at T2. There was a significant difference in ankle evertor strength between the two groups at T1 and T2 (*P* = 0.032 and *P* = 0.024). Significant interaction effects were not observed in ankle dorsiflexor or plantar flexor strength after the intervention.

The time and group interaction effects on the spatiotemporal gait metrics, including the preferred walking velocity, cadence, step length, stride length, ratio of stance/swing phase, and double stance phase duration as a percentage of a gait cycle, were not significant after the intervention. However, the preferred walking velocity, cadence, step length of the affected side, and double stance phase duration as a percentage of a gait cycle tended to improve in both the intervention group and the control group after the intervention.

^a^ *P* value of a time and group interaction effect between the two groups by repeated measures analysis of variance. ^b^Analysis of within-group changes over time by paired *t*-test. ^∗^*P* < 0.05.

## 4. Discussion

Hospital-based rehabilitation therapy for 12 weeks relieved pain and symptoms and improved patients' independence in ADL, sports activity levels, and QOL more effectively than did education-based rehabilitation at home in post-MBP patients. In addition, these effects lasted for up to 1 month after the intervention ended. We have confirmed that appropriate rehabilitation in post-MBP CAI patients is important to improve ankle function and QOL in the early postoperative period, although successful surgical treatment is essential.

The rehabilitation protocols for lateral ankle ligament operative patients vary considerably because no specific rehabilitation guidelines for these ankle instability surgeries have been established yet [20, 21]. Although the importance of postoperative rehabilitation is recognized by both patients and orthopedic surgeons, in South Korea, rehabilitation has usually been performed by the patients at home after they participate in an educational session. In some cases, private clinical therapists who lack an understanding of the intentions of surgical doctors and surgical treatments are responsible for educating patients on how to perform rehabilitation exercises after the MBP. CAI patients may experience complications such as pain or reinjury of ligaments or can be dissatisfied with the poor recovery of ankle function, despite the surgical treatment being sucessful [9]. Patients can receive rehabilitation therapy customized to their functional level and close monitoring by therapists collaborating with orthopedic surgeons through hospital-based rehabilitation. In this study, the patients who received hospital-based rehabilitation showed excellent compliance, which is assumed to be an important reason for better ankle recovery after the MBP in the intervention group than in the conventional education-based rehabilitation therapy group [22].

The post-MBP rehabilitation protocol in this study included functional rehabilitation, joint range of motion exercises, and strengthening exercises. The functional rehabilitation therapy was composed of balance training and proprioception facilitation exercises. These exercises enhanced the balance and strength of the ankle joint by simultaneously involving a balance board [23]. Additionally, coordination exercises, such as single-limb standing exercises, are usually administered to improve proprioception and ankle strength [24]. We administered rehabilitation therapy to the patients who were capable of standing with full weight-bearing without pain. Joint exercises and ankle strengthening exercises are essential to facilitate ankle function restoration and to prevent recurrence [25, 26]. These exercises have a larger effect when they are performed in a facility rather than at home [9]. The hospital-based rehabilitation protocol used in this study was a protocol that did not differ significantly from other protocols. However, we assumed that sufficient therapeutic intensity due to high compliance promoted patient recovery.

Hospital-based rehabilitation improved ankle evertor and invertor muscle strength more effectively than self-rehabilitation at home. Ankle muscle weakness has been regarded as a potential cause of CAI; however, there is limited research on ankle motor function characteristics in CAI patients. Several previous studies revealed that evertor and invertor weakness was obvious in CAI patients, although the strength of the ankle dorsiflexor and plantar flexor was relatively preserved [27, 28]. These results are similar to those of our study, and the participants in both the intervention and control groups showed prominent ankle evertor/invertor weakness. To restore ankle motor strength, strength training has typically been an integral part of the CAI rehabilitation process [27]. Strength-training exercises are often initiated in combination with balance and proprioception training as soon as a pain-free range of motion is achieved and resistive forces can be tolerated [27, 28]. Although these exercises were included in both protocols in this study, we assumed that greater compliance with the hospital-based rehabilitation program and the physical therapists played a positive role in improving ankle evertor and invertor strength. Because the ankle dorsiflexor and plantar flexor strengths were preserved similar to those of the unaffected side, these muscle strength improvements were relatively insignificant in this study.

Gait indicators such as the preferred walking velocity, cadence, step length of the affected side, and double stance phase duration as a percentage of a gait cycle tended to improve. However, there were no significant differences between the two groups. This intervention was conducted on ATFL grade 2 or 3 patients. The results of the preintervention gait analysis did not actually show any specific abnormalities. In contrast to our results, Anandacoomarasamy and Barnsley [29] reported that gait metrics such as velocity, cadence, step length, the base of support, stance, and single-limb support were abnormal in CAI patients. However, the severity was unknown in that study, so that we assumed that the participants might have been more severe cases. Previous studies have suggested several methods to evaluate the gait function of CAI patients more accurately. For example, the modified backward walking test or faster treadmill speed test could more clearly reflect walking ability in the CAI patients than conventional gait analysis [30, 31]. The use of these methods should be considered for future trials for CAI patients.

This study has several limitations. The patients who participated in the hospital-based rehabilitation program had a short follow-up period. Long-term postoperative ankle function and patient activity levels could not be confirmed in this study. Additionally, we were not able to determine whether conservative education-based rehabilitation at home had been conducted appropriately by each patient. After patients learn to perform the rehabilitation exercises in an outpatient session, patients may perform the exercises differently, depending on their understanding and compliance levels. Although we checked that the patients in the control group were performing the exercises once a month, we could not ensure they were performing them appropriately. These limitations should be considered when interpreting the results.

## 5. Conclusions

In summary, rehabilitation after the MBP plays a vital role in the recovery of normal ankle function in CAI patients. Hospital-based rehabilitation, administered by physical therapists and physiatrists, more effectively improved postoperative pain, uncomfortable symptoms, independence in activities of daily living, sports activity levels, and QOL than did education-based rehabilitation, which was performed independently by the patients at home. Therefore, it is recommended that a systemic hospital-based rehabilitation protocol is implemented in collaboration with a rehabilitation team for postoperative CAI patients.

## Figures and Tables

**Table 1:** Baseline clinical characteristics.

	Intervention group (*n* = 18)	Control group (*n* = 17)
Age (years)	39.8 ± 6.9	40.5 ± 7.1
Gender (M : F)	10 : 8	9 : 8
Height (cm)	169 ± 11	168 ± 10
Weight (kg)	69.1	70.0
BMI (m^2^/kg)	21.2 ± 2.9	22.0 ± 3.8
Affected side (Rt : Lt)	10 : 8	8 : 9
Arthroscopic grade		
Grade0	0	0
Grade 1	0	0
Grade 2	8	7
Grade 3	10	10
Grade 4	0	0
Duration of illness before MBP (months)	6.5 ± 2.0	6.7 ± 1.8

Data are presented as the mean ± SD. MBP = modified Broström procedure; BMI = body mass index.

**Table 2:** Comparison of the foot and ankle outcome score between the intervention and the control group.

	Intervention group (*n* = 18)			Control group (*n* = 17)			*P* value^a^
	T0	T1^b^	T2^b^	T0	T1^b^	T2^b^	
Pain	70.4 ± 7.6	85.3 ± 10.2^∗^	92.5 ± 11.2^∗^	71.4 ± 8.5	77.2 ± 8.4^∗^	77.1 ± 8.3	0.029^∗^
Symptoms	70.9 ± 9.3	86.1 ± 12.5^∗^	92.4 ± 13.3^∗^	72.2 ± 8.3	77.1 ± 9.0^∗^	77.0 ± 9.9	0.044^∗^
ADL	67.5 ± 7.0	81.6 ± 9.5^∗^	89.6 ± 10.0^∗^	66.5 ± 5.0	73.3 ± 8.2^∗^	73.4 ± 7.5	0.047^∗^
SRA	53.4 ± 7.3	85.0 ± 12.8^∗^	95.1 ± 14.2^∗^	53.8 ± 4.9	62.5 ± 6.5^∗^	62.8 ± 7.7	0.008^∗^
QOL	58.2 ± 6.8	78.9 ± 9.1^∗^	88.3 ± 12.0^∗^	60.0 ± 6.1	71.7 ± 7.0^∗^	73.7 ± 9.4^∗^	0.048^∗^

ADL = activities of daily life; SRA = sports and recreational activities; QOL = quality of life. ^a^*P* value of a time and group interaction effect between the two groups by repeated measures analysis of variance. ^b^Analysis of within-group changes over time by paired *t*-test. ^∗^*P* < 0.05.

**Table 3:** Comparison of the ankle strength of the affected side and spatiotemporal gait metrics between the two groups after the intervention.

	Intervention group (*n* = 18)			Control group (*n* = 17)			*P* value^a^
	T0	T1^b^	T2^b^	T0	T1^b^	T2^b^	
Ankle motor strength (nm)							
Invertor	20.5 ± 9.4	23.1 ± 9.5^∗^	25.2 ± 10.1^∗^	21.1 ± 9.6	22.1 ± 10.6^∗^	22.2 ± 10.4	0.047^∗^
Evertor	16.1 ± 5.4	17.6 ± 6.1^∗^	18.0 ± 7.5^∗^	15.8 ± 6.0	15.9 ± 6.1	16.0 ± 6.3	0.044^∗^
Dorsiflexor	22.5 ± 7.4	22.3 ± 8.0	22.6 ± 7.5	23.0 ± 6.9	22.8 ± 8.4	23.1 ± 8.1	0.682
Plantar flexor	39.4 ± 11.5	40.1 ± 13.5	40.2 ± 12.9	40.0 ± 13.1	39.5 ± 12.0	40.5 ± 12.3	0.540
Gait analysis							
Velocity (km/h)	3.2 ± 1.1	3.9 ± 1.3^∗^	4.1 ± 1.4^∗^	3.3 ± 1.2	3.5 ± 1.3^∗^	3.7 ± 1.4^∗^	0.384
Cadence (steps/min)	95 ± 12	105 ± 16^∗^	109 ± 16^∗^	93 ± 13	102 ± 14^∗^	107 ± 17^∗^	0.190
Step length, affected (cm)	48 ± 7	55 ± 8	56 ± 8	48 ± 9	52 ± 9	55 ± 9	0.581
Step length, unaffected (cm)	55 ± 8	56 ± 8	56 ± 9	56 ± 9	57 ± 9	56 ± 9	0.736
Stride length (cm)	100 ± 14	105 ± 15	110 ± 16	99 ± 15	106 ± 17	111 ± 17	0.759
Stance phase, affected (%)	64 ± 12	64 ± 12	65 ± 13	65 ± 12	63 ± 13	64 ± 12	0.610
Stance phase, unaffected (%)	65 ± 12	63 ± 13	64 ± 13	64 ± 12	65 ± 12	65 ± 12	0.474
Swing phase, affected (%)	36 ± 8	36 ± 9	35 ± 8	35 ± 8	37 ± 8	36 ± 8	0.399
Swing phase, unaffected (%)	35 ± 8	37 ± 8	36 ± 9	36 ± 8	35 ± 7	35 ± 7	0.581
Double stance phase (%)	34 ± 7	31 ± 6^∗^	29 ± 6^∗^	33 ± 7	31 ± 6^∗^	31 ± 6	0.402

## Data Availability

The datasets analyzed during the current study are available from the corresponding authors on reasonable request.
